# Benzodiazepines in sport, an underestimated problem: Recommendations for sports medicine physicians’ practice

**DOI:** 10.3389/fpsyt.2022.1066330

**Published:** 2022-12-21

**Authors:** Thomas Zandonai, Ana María Peiró, Francesca Fusina, Fabio Lugoboni, Lorenzo Zamboni

**Affiliations:** ^1^Department of Pharmacology, Paediatrics and Organic Chemistry, Miguel Hernández University of Elche, Alicante, Spain; ^2^Neuropharmacology on Pain and Functional Diversity, Institute of Health and Biomedical Research of Alicante (Instituto de Investigación Sanitaria y Biomédica de Alicante), Alicante, Spain; ^3^Department of Social and Developmental Psychology, Sapienza University of Rome, Rome, Italy; ^4^Pain Unit, Department of Health of Alicante, University General Hospital, Alicante, Spain; ^5^Clinical Pharmacology Unit, Department of Health of Alicante, University General Hospital, Alicante, Spain; ^6^Padova Neuroscience Center, University of Padova, Padua, Italy; ^7^Department of General Psychology, University of Padova, Padua, Italy; ^8^Unit of Addiction Medicine, Department of Internal Medicine, Integrated University Hospital of Verona, Policlinico “G.B. Rossi”, Verona, Italy

**Keywords:** addiction, sleep, insomnia, anxiety, drugs, guidelines

## Abstract

In the last years, only few studies in literature have focused on the use and abuse of benzodiazepines (BZDs) in sport. Benzodiazepine-related problems include misuse, addiction, driving impairments, and morbidity and mortality related to overdose and withdrawal. Two clinical cases regarding elite endurance athletes evidenced that they had started to use BZDs to counteract insomnia, to recover faster from training sessions and to manage muscle pain. One of the important points that emerged from their stories was that their sports doctors did not recognize the drugs’ addictive properties, and did not intervene to gradually reduce the dosage. Experts have previously provided recommendations for BZD therapy management in clinical practice. In this article, we would like to address sports medicine physicians specifically and provide guidelines to help them manage situations involving BZD prescription, the recognition of addiction, and intervention strategies.

## Introduction

Sleep is vital for health and well-being and it is important for cognitive functioning, mood, mental health, and cardiovascular, cerebrovascular, and metabolic health (1). The need to guarantee adequate sleep to elite athletes has increasingly prompted researchers to investigate the most important factors influencing athletes’ sleep quality. Recent studies have addressed the effects of overtraining during preparation (2) and the benefits of interventions that both assess and manage travel fatigue and jet lag (3). Additionally, these studies proposed strategies for enhancing sleep quality as well as tools for practitioners who manage and optimize athletes’ sleep (2, 3).

Poor sleep may diminish athletic performance, impair recovery, and increase the risk of injuries (2, 4). Moreover, it compromises athletes’ abilities to maintain both good performance levels and a positive mood. A possible risk factor for sleep disturbances in athletes is also anxiety (2, 5, 6). Indeed, a recent meta-analysis on current elite athletes showed that 34% of them had symptoms of anxiety/depression (7). For this reason, occasionally, the loss of this balance could induce them to turn to sleeping medications as a solution. Over the past few years, research has tried to direct the attention of the sporting community to the use and abuse of benzodiazepines (BZDs) in sport (8–10).

Benzodiazepines are among the most commonly prescribed medications for insomnia and anxiety, and they are extensively used in clinical practice. BZDs act as positive allosteric modulators of the GABA-A (Gamma-Aminobutyric Acid Type A) receptor (11). BZDs can be subdivided into different groups based on their chemical structure and pharmacokinetic properties, resulting in different associated mechanisms of action and consequent clinical effects. Long-term BZD use is generally avoided due to their potential in the development of addiction (12, 13). Indeed, several studies have evidenced that BZDs should be considered a suitable treatment for specific clinical situations and for short-term use only (2–4 weeks) (14).

Despite clinical recommendations, long-term BZD users range from 6 to 76% of total users. Fifteen to forty-four percentage of them present moderate-to-severe withdrawal symptoms, and 3–4% have a full-fledged addiction (15). These drugs present several dangerous side effects. Among these, some of the most important are: multifocal cognitive dysfunction (16); the fact that BZDs could impair information acquisition, with additional adverse effects on anterograde memory processes (17); and, finally, the increase in cognitive decline incidence in the elderly, especially when they make long-term use of BZDs (18, 19). Regarding driving, a recent study showed that the impairing effects of benzodiazepine hypnotics on driving may mitigate over time following long-term use (i.e., 3 years or more), although the BZD-related neurocognitive impairments may remain (20). BZDs inhibit transmission on the postsynaptic γ-aminobutyric acid (GABA) neurons inducing a decrease in muscle spasms through alterations of central nervous system conduction. One of their effects is induced muscle weakness, which could put serious strain on the joints and back especially during intensive sport efforts, causing possible injuries. However, quality studies on this issue are needed to support this (21, 22).

Moreover, BZDs used in association with other drugs could increase difficulties in treatment (23); BZD use is associated with increased risk of car accidents (24); long- and short-acting BZDs increase the risk of falling, especially in elderly patients and children (25–27), as well as accident-related consequent hospital admissions (28, 29). In pregnant women, BZDs could contribute to neonatal morbidity and some congenital malformations (30). Lastly, long-term BZD use has been found to reduce the quality of life (31).

### Benzodiazepines in sport

A few years ago, some studies tried to encourage the debate on the use of BZDs in sports (32, 33), but only in recent years does interest seem to have been rekindled. Indeed, two clinical cases were reported in literature regarding elite endurance athletes, both addicted to such high doses of BZDs that they needed detoxification even after a week of hospitalization (8, 9) using a slow infusion of flumazenil (Verona approach) (34). The first of the two cases was that of a 38-year-old professional cyclist. He used high doses of caffeine, and subsequently cocaine, to improve his performance. This led him to develop insomnia, which he tried to manage with lormetazepam, to which he became addicted; furthermore, he continued his caffeine and cocaine use, especially in the morning, to counteract the effects of lormetazepam, in what became a vicious cycle of addiction (8). His medical history showed long-term lormetazepam use in the form of an oral solution, starting from 2.5 mg/ml and leading to 20 ml 1/4 50 mg, the equivalent of 250 mg of diazepam per day.

In the second case, a 30-year-old female elite marathon runner was similarly in treatment for lormetazepam detoxification. She too reported increasing her daily intake of lormetazepam to sleep better and to increase her training performances. As a result, her lormetazepam intake reached a total of 360 ml (900 mg) of lormetazepam per day, which is the equivalent of 4500 mg/day of diazepam.

According to their anamnesis, they had started to use lormetazepam to counteract insomnia, to recover faster from exercise sessions and to manage muscle pain. Regarding this last issue, studies which investigated the effects of BZDs on pain management found limited utility and conflicting results (35–37). In addition, athletes likely experience pain and pain treatment differently than people undertaking general exercise, and they may be more likely to use different types of analgesic drugs, incurring in more risks than benefits (38, 39). Like all medications, BZDs have the potential for both harm and benefit. For this reason, physicians should help patients consider these factors appropriately, and develop a treatment plan that is safe and effective for them (40). However, one of the most striking points that emerged from these two cases was that their sports doctors did not recognize the drugs’ addictive properties, and no intervention to reduce the therapy was implemented.

Given that there is a widespread conviction among athletes that taking drugs will improve their performance, the question arises whether there is evidence that BZDs, which are not prohibited in sport, are indeed beneficial to exercise performance. According to our latest literature review, it seems that BZDs have no ergogenic effect on exercise performance and potentially even have a negative effect on exercise. The ten studies reviewed by the authors used relatively small sample sets of participants and heterogeneous methodologies with regards to exercise test type, participant type, BZD types and the doses administered. The therapeutic use of BZDs seems unquestionable, but another source of uncertainty is that most of the studies failed to report if BZDs actually improved sleep quality or reduced pain. The review highlights that the drugs’ active mechanisms are still unknown, that further studies are needed in order to increase the number of participants, and that female participants must be included to discern gender’s influence on the drugs’ effects (10). Moreover, the risk of misuse is still largely underestimated by clinicians and institutions around the world and chronic users of BZDs may develop deficits in working memory, learning, and attention. Depression, injurious falls, and traffic accidents are other common complications related to chronic use of BZDs (12).

### Recommendations for sports medicine physicians

The above-mentioned data are required in order to enable the creation of evidence-based policies and guidelines for treatment. Experts have previously provided recommendations for the management of benzodiazepine therapies in the clinical practice (14, 41), but we would like to make the following recommendations to sports medicine physicians specifically to help them in future situations which entail benzodiazepine prescription, the recognition of addiction, and intervention strategies.

Sports medicine physicians should:

1. examine the specific benzodiazepine’s likely benefits and risks in each individual case early in treatment and, in the case of prescription, closely monitor any behavior which could indicate misuse/abuse;
2. have knowledge concerning any concomitant use by the athlete of other drugs (such as analgesics) or non-pharmacological treatments, if necessary;
3. evaluate alternative treatments to benzodiazepines, especially if the treatment is expected to last more than a month;
4. we also suggest that they conduct a complete psychological assessment with an accurate anamnesis in order to ensure prescription of the best treatment for the patient;
5. calculate the potential duration of the treatment well in advance, considering any need for long-term drug therapy for insomnia and anxiety disorders; consider conducting psychological evaluations and multimodal medical assistance;
6. maintain close contact with patients that use BZDs and constantly monitor them to promptly address misuse/abuse behavior, specifically checking whether the patient:
- takes therapeutic (low) doses for more than 3 months;
- needs to take benzodiazepines to carry out normal sports activities every day;
- continues using the drug although the original therapeutic indication has ceased to be necessary;
- has difficulty stopping use of the drug or reducing the dosage due to withdrawal symptoms;
- develops symptoms of anxiety between doses or, in the case of short-term benzodiazepine use, develops cravings for the next dose;
- maintains regular contact with his/her sports medicine physicians to request repeated prescriptions;
- displays elevated anxiety levels if the next prescription is not fulfilled rapidly;
- increases their dosage compared to the original prescription and if they ask for prescriptions more frequently;
- shows continued symptoms of anxiety, panic, agoraphobia, insomnia, depression, or physical symptoms despite the extended use of benzodiazepines;
7. gradually reduce the drug dosage after introducing a more specific therapy;
8. reconsider the diagnosis if the patient does not respond to therapy, or if the drug is taken for a longer duration or at higher doses than were originally foreseen.
9. It is important to remember that benzodiazepines are generally safe drugs, but that they could become very dangerous if combined with other substances, such as alcohol or opioids.

Recently, the International Olympic Committee (IOC) and the National Collegiate Athletics Association (NCAA) have addressed sleep as a major contributor to athletic performance and as a fundamental feature of athletes’ mental health. These statements represent the increased awareness of the importance of sleep health among athletes (42).

## Conclusion

In this article, we would like to address sports medicine physicians specifically and provide guidelines to help them manage situations involving BZD prescription, the recognition of addiction, and intervention strategies. We believe that these recommendations could also help to athletes and warn them of the possible effects that BZD can induce if taken without medical supervision.

Finally, it is important that sports medicine physicians keep in mind that BZDs are potentially addictive, particularly fast-acting BZD typologies. Therefore, due to the lack of evidence about clinical issues regarding misuse criteria, additional information about the complicated relationship between BZDs and exercise is required, possibly involving larger samples.

## Data availability statement

The original contributions presented in this study are included in this article/supplementary material, further inquiries can be directed to the corresponding author.

## Author contributions

TZ and LZ substantially contributed to the conception, drafting of the manuscript, and critical revision of the manuscript for important intellectual content. All authors reviewed the manuscript, provided significant input, read, and approved the submitted version.
